# False Impressions

**Published:** 1888-11-17

**Authors:** Caroline Fothergill

**Affiliations:** Author of "An Enthusiast," "A Voice in the Wilderness," etc.


*(To be continued.)*

### False Impressions.

By Caroline Fothergill, Author of "An Enthusiast," "A Voice in the Wilderness," etc.

*(Continued from p. 94.)*

### Chapter VII.—The Flower Show.

The Mayfield Chrysanthemum Show was an annual event, the closing one of a series of flower shows which were held all through the spring and summer down to early winter. People subscribed to the whole series, and the ground in which the shows were held was considered one of the fashionable promenades of Mayfield. This present show, being in winter, was under cover; but the cover was only glass, both sides and roof were glass; the roof was almost hidden with creepers, and trailing plants, and hanging fern-baskets; but the sides were clear, allowing glimpses of the beautiful grounds outside, except where the tiers of flowers rose too high. The building was a long kind of avenue, terminating in a vast round daïs in the middle of from which a fountain played and the band sat. This avenue had various ramifications, all furnished with exquisite hot-house plants and ferns, and amply provided with seats and lounges.

Everyone was there, all the youth and beauty and fashion of both sexes of Mayfield, and everyone seemed to know everyone else; only Edith knew no one but herself and her companion.

They walked about for some time; Edith, filled with delight at the flowers and the people, George looking after her, and raising his hat every minute to people he knew, until Edith was quite impressed with the extent of his acquaintance. After a while George suggested that they should sit down, and they went to a quiet nook, from which they could see all that was going on, and the noise of the band was not too loud. Here they found seats, and began to talk. This was rather a nervous time for Edith; so long as she had only had to admire and have things explained to her she had been quite content, even happy; but when it became evident that George intended not only to talk to her, but to make her talk to him, her content vanished, and she began to feel nervous and ill at ease.

His first question was one which almost every man would have put under the circumstances. He looked down at the slender figure by his side and asked:

"Well, are you enjoying yourself?"

"Oh yes, thank you, so much," was her eager answer. "It was so kind of you to bring me, only I am afraid ——"

"Afraid of what?" as she paused.

"I mean," she went on after a little hesitation, "I should be enjoying myself, but ——"

She paused again, and he felt half amused, half bored, for fragmentary conversation of this kind was not to his taste. He had had no experience of shy young girls, and he did not quite know how to accommodate himself to them.

"What is it you are trying to say?" he asked at length, not sharply, but a little abruptly, as he often spoke.

Edith blushed more than ever, but desperation and a sudden fear that she was becoming "troublesome," urged her on.

"I remember," she said, "that you said you did not want to come, and I didn't like to think that you came just for me."

He laughed; yes, positively laughed, and she scarcely knew whether to feel relieved; she had been afraid he might be angry, but it was almost worse to be laughed at. Then he turned upon her with rather a quizzical look.

"Upon my word," he began, and then concluded, "How do you know I came here just for you?"

Edith's heart seemed to withdraw completely from her body, and she was filled with burning shame and embarrassment. What had she said? What a conceited goose he must have thought her! Was it in the least likely that he would alter his arrangements and come out here just to please her, a girl to whom he had scarcely ever spoken, of whom he never took any notice? Acting on the impulse roused by his first words, she had decided that when they should be alone, and a favourable opportunity should present itself, she would express her gratitude for his kindness, and her regret that he should have disturbed himself on her account. Suddenly the matter was presented to her in a totally new light, the tables were completely turned upon her; she was too shy to say any more, and could only sit silent, blushing and miserable.

George saw her trouble, and it suddenly flashed upon him that here was a very delicate little girl indeed, who could not bear teazing, or any treatment except the most gentle and considerate. His heart—this "morbid cynic" had a heart—smote him, and, acting as impulsively as she herself could have done, he laid his hand upon her arm, and said—

"My dear child, don't look so distressed; you make me feel as if I had committed a murder. I did come on purpose to please you, but there was no compulsion. As soon as you said you wanted to come I began to want to come too; and now I am here I am enjoying myself very much. Will that do?"

He smiled as he spoke—the smile which made even Beatrice Stanford think him attractive, and which affected Edith as the sunshine affects a plant. It did not seem to her as if it were winter. In this warm, flower-scented atmosphere, with the swinging, dreamy music coming by snatches to her ear, she felt as if she were in Paradise; she forgot everything except the present moment, which she felt was very good.

"I believe," went on George, as she said nothing, but only smiled shyly in answer to his last words, "that you knew all along that I was enjoying myself; but you are so exacting that you could not be satisfied until you had wrung the confession from me."

"Oh, Mr. Murray!" said Edith, horrified out of her shyness at such conduct being attributed to her.

"It is pleasure enough to see your pleasure, child. You are so fresh and young. You have seen so little that even a flower-show is a new and exciting experience to you."

"I know I have seen very little," said Edith, "but I never thought any one would think it a good thing. I have always thought people would despise me for my ignorance, and that I should be left in a corner by myself. I have often been afraid to speak to other girls, they knew so much more than I did."

The string of her tongue was loosened, both George and herself noticed it, though perhaps neither could have clearly explained what had brought about the change. George began to find the conversation interesting.

"You will not be left in a corner," he said encouragingly, "unless by people who have no sense of refinement or delicacy, and you will not care to be sought by such people. When you reproach yourself for being ignorant, you are perhaps also ignorant of the fact that most girls nowadays know a great deal too much, and that innocence and freshness like yours are very charming."

She did not reply; she could think of nothing to say. George only wanted to cheer her up, and give her a little more self-confidence than she had now, but he had succeeded in completely overwhelming her and putting her thoroughly out of countenance. Before the silence had become awkward, the band suddenly ceased playing, the men rose from their seats, and there was a general surge of people in one particular direction.

George roused himself, and looked round. "It is two years since I was here," he said; "but if I am not mistaken that used to be the signal for tea. What do you say to tea, Edith? Would you like some?"

During the foregoing conversation, Edith had been entirely and very comfortably oblivious of everything in the world except her immediate surroundings. This sudden question from George brought back with a rush the recollection of Mrs. Lester, with all her restrictions and instructions, especially as they bore upon this very matter of tea. The mention of it, and the sight of so many people hurrying away in search of the refreshing cup, caused her to realise that she was very thirsty, and would have enjoyed nothing more than some tea. But she dared not disobey her aunt on such slight provocation as a mere suggestion, so she answered, although not without hesitation—

"Oh, thank you, I don't know; no, thank you, I think not."

"Not!" echoed her companion. "I thought that if women did not get tea at four o'clock they all withered up like flowers in want of rain."

Edith smiled constrainedly, and looked a little awkward.

"No, thank you," she repeated, "I don't think I want any."

George was silent for a moment, during which he was visited by an inspiration. He could not have told what put it into his head, but he all at once felt convinced that his sister was at the bottom of Edith's refusal. He made no further effort to persuade her, merely saying within himself: "This is Frances' doing, I am certain. She bullies the girl; she used to try to bully me, and I consider it my duty to circumvent her and to frustrate her knavish tricks."

Aloud he said:

"Well, I am very thirsty, but I can't leave you here while I go and refresh myself. Will you, since I can't persuade you to have any tea yourself, at least come with me and bear me company while I have a cup?"

She was rather disappointed that he was so easily convinced she would not have any, although if he had gone on urging her, she would have become painfully embarrassed; but she at once consented, and they went off to the tea-room. Just as they were entering it they met two ladies coming out. One was tall and very beautiful, with a proud, sweet face, and a dignified carriage. The other was a tiny woman, with a bright, clever face, and eyes which looked as if they took in a whole world at a glance. Both women were, as Edith remarked at once, exquisitely dressed. As they met, the younger lady bowed gravely and slightly to George; he lifted his hat with equal gravity, equal courtliness. The little lady swept her brilliant eyes over the whole group, and they were gone, leaving George with a frown on his brow.

"Who were those ladies, Mr. Murray?" asked Edith, who had been much impressed by the little scene, the graceful dress of the women, and the ceremony of the salutes which had passed between one of them and her companion.

"The taller one, who bowed to me, is called Miss Beatrice Stanford. I do not know the lady with her," was his reply.

"She was very beautiful," said Edith timidly, for George's tone was not the same as it had been when they sat in that quiet nook.

"Yes, she has a beautiful face," he said, as he had said once before.

"Do you mean it is only her face which is beautiful?" asked the girl thoughtfully; and George looked down at her with a smile.

"What an observant little girl you are," he said. "Yes, I did partly mean that. Miss Stanford's face is very beautiful, and as she wears no veil everyone is at liberty to see it, but her mind is more inscrutable."

"I think she looks as if she were good," with more decision than she usually showed.

"I quite agree with you," said George, rather drily, and the subject dropped.

After tea, of which it is needless to say Edith partook as well as George, they had another stroll among the flowers, and then drove home again. Edith, full of new thoughts and feelings which she did not understand, conscious that she had disobeyed every one of her aunt's directions, and with an utterly unaccountable feeling that she did not care if she had.

"So that is your phantom," said Laura, when she and Beatrice were again at home. "This is the first time I have seen him face to face, and I like him. He does you credit; he has decidedly the grand air. I was disappointed when you only bowed; I thought you would have spoken."

Beatrice shrugged her shoulders.

"There was nothing to speak about. We never talk of anything except matters connected with the Museum."

"Dear me!" with affected innocence, "that must be very dull. I fancied you were on quite intimate terms."

"Oh, no! you were quite mistaken."

"I see I was," said Mrs. Ledward rather dryly. "I wonder," she went on, "who that girl was with him. She was a pretty little thing, and the contrast between them was comical. He looked like a prince in disguise, and she was decidedly shabby."

"I saw no girl," said Beatrice, who spoke as though she were unwilling to continue the subject.

"Where were your eyes? I saw her at once, and wondered who she was. There was an air of fatherly solicitude in the way in which he looked after her which in a man of his years was decidedly laughable," and she laughed herself as she spoke.

"How you exaggerate," said Beatrice crossly. "It was impossible you should see all that in the mere second we were in passing them."

"I had seen them coming from afar. I had also been watching them earlier in the afternoon, though I was not quite sure it was Mr. Murray, and so said nothing of it to you. I wonder if she is his ward. Wards of that age are very awkward."

